# C-176 loaded Ce DNase nanoparticles synergistically inhibit the cGAS-STING pathway for ischemic stroke treatment

**DOI:** 10.1016/j.bioactmat.2023.07.002

**Published:** 2023-07-18

**Authors:** Zhixin Zhu, Haipeng Lu, Lulu Jin, Yong Gao, Zhefeng Qian, Pan Lu, Weijun Tong, Pik Kwan Lo, Zhengwei Mao, Haifei Shi

**Affiliations:** a Department of Orthopedics, 1st Affiliated Hospital of Zhejiang University School of Medicine, Qingchun Road 79, Hangzhou, 31000, China; b MOE Key Laboratory of Macromolecular Synthesis and Functionalization, Department of Polymer Science and Engineering, Zhejiang University, Hangzhou, 310027, China; c Department of Chemistry and State Key Laboratory of Marine Pollution, City University of Hong Kong, Tat Chee Avenue, Kowloon, Hong Kong

**Keywords:** Ischemic stroke, Ce-based nano-nuclease, The cGAS-STING signaling pathway, Anti-inflammation

## Abstract

The neuroinflammatory responses following ischemic stroke cause irreversible nerve cell death. Cell free-double strand DNA (dsDNA) segments from ischemic tissue debris are engulfed by microglia and sensed by their cyclic GMP-AMP synthase (cGAS), which triggers robust activation of the innate immune stimulator of interferon genes (STING) pathway and initiate the chronic inflammatory cascade. The decomposition of immunogenic dsDNA and inhibition of the innate immune STING are synergistic immunologic targets for ameliorating neuroinflammation. To combine the anti-inflammatory strategies of STING inhibition and dsDNA elimination, we constructed a DNase-mimetic artificial enzyme loaded with C-176. Nanoparticles are self-assembled by amphiphilic copolymers (P[CL_*35*_-*b*-(OEGMA_*20.7*_-*co*-NTAMA_*14.3*_)]), C-176, and Ce^4+^ which is coordinated with nitrilotriacetic acid (NTA) group to form corresponding catalytic structures. Our work developed a new nano-drug that balances the cGAS-STING axis to enhance the therapeutic impact of stroke by combining the DNase-memetic Ce^4+^ enzyme and STING inhibitor synergistically. In conclusion, it is a novel approach to modulating central nervus system (CNS) inflammatory signaling pathways and improving stroke prognosis.

## Highlights

• We synthesized C176 loaded Ce(IV)- artificial DNase.
• The NTA/Ce(IV)/C-176 nanoparticles achieve dsDNA scavenging and STING inhibition.
• The NTA/Ce(IV)/C-176 nanoparticles effectively alleviate the immune inflammation of stroke and save neurons.

## Graphical abstract

**Figure:**
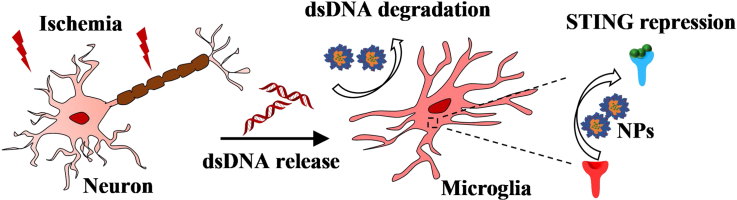

## 1 Introduction

**Scheme 1:**
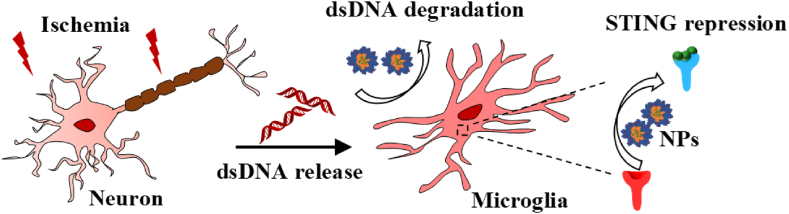
The therapeutic mechanism of NTA/Ce^4+^/C-176 nanoparticles.

Ischemic stroke is a complicated CNS disorder caused by the occlusion of cerebrovascular, with a high rate of morbidity and mortality [1]. Although the effectiveness of clot dissolution and clot retrieval in a therapeutic time window has been proven in clinical trials, only 7% of all stroke victims benefit from these procedures [2,3]. The devastating condition of cutting off oxygen and glucose to brain parenchyma over 4.5 h results in irreversible nerve cell death, releasing cell free-double strand DNA (dsDNA), which would be as a kind of damage-associated molecular patterns (DAMPs) to initiate innate immune signaling cascade and promote chronic inflammation [4]. There is a pressing need for anti-inflammatory therapeutics to protect neurons from durable damage (see Scheme 1).

The neuroimmune microenvironment encompasses a wide array of central and peripheral immune cells and remains a promising target for the treatment of a variety of disorders. Microglia, the CNS-resident macrophages, are the primary intracerebral surveillants to maintain neuro-microenvironment homeostasis by migrating to the site of pathology for phagocytosis of DNA fragments emanating from the post-injury area [5,6]. Targeting microglia is a desirable therapeutic approach for altering the post-injury microenvironment [[7], [8], [9]]. The ischemic cell debris in situ contains amounts of DNA segments released from damaged nerve cells and apoptotic immune cells [10]. Furthermore, the expression of deoxyribonuclease (DNase) is insufficient [11,12], resulting in an aberrant buildup of free dsDNA in the ischemic zone [13,14]. The excessively accumulated dsDNA segments from ischemic tissue debris are engulfed by microglia and detected by their cyclic GMP-AMP synthase (cGAS) [15,16] to trigger robust activation of the innate immune stimulator of interferon genes (STING) pathway, which has emerged as a powerful driver in persistent post-injury neuroinflammation [[17], [18], [19], [20]]. STING has been shown to be mostly overexpressed in microglia as opposed to neurons or astrocytes [21], and the up-regulation of STING impaired neurological performance and cognitive functions [22]. Preventing cGAS-STING activation after ischemic stroke helps alleviate neuroinflammatory burden [[23], [24], [25]].

Only blocking the chronic activation of STING is insufficient [26] if we do not clear immunogenic dsDNA that constantly activates cGAS. It has been studied that dsDNA longer than 45 bp strongly activated human cGAS (hcGAS) [27,28]. Because dsDNA comes into a variety of sequences, geometries, and modifications, it is challenging to selectively eliminate these polynucleotide fragments via sequence discrimination or structural interactions [29]. Recently, cationic polymers have been applied to electrostatically neutralize negatively charged dsDNA to attenuate the proinflammatory signaling cascade [[30], [31], [32], [33], [34], [35], [36], [37], [38]]. Liang et al. prepared a block polymer with the cationic poly(2-(diethylamino)ethyl methacrylate) (PDMA) shell for scavenging dsDNA [30]. Wu et al. tuned its cationic ratio by mixing poly(ethylene glycol) (PEG) into the PDMA shell because the highly positive charges would cause hazardous systemic toxicity [32]. Polyamidoamine (PAMAM) [31], polyethyleneimine (PEI) [33,34,37], and diethylamino group modified polydopamine [36] were also applied as the nucleic acid binding cationic sites, which showed strong adsorption capability of negative charges but with no discrimination. Even though the DNA was tightly restricted by anionic materials, it has been determined that the half-life of phosphodiester groups in DNA under physiological circumstances (pH 7, 25 °C) is 200 million years with a high hydrolysis resistance when just the hydrolytic breakage of the P–O bond is taken into account [39]. It could be advantageous to use a therapeutic DNase that consumed dsDNA continuously and did not have a high density of cationic alteration. Enzymes have been proven to be viable therapeutic agents. Laridan et al. found that DNase I improved the effectiveness of t-PA for *ex vivo* lysis of patient thrombi [40]. Additionally, DNase I has been applied to conjugate with microgels [41] and nanoparticles [42,43] to attenuate nucleic acid-induced inflammation.

Nevertheless, the drawbacks of conventional biological enzymes, such as their elevated cost, poor stability, and limited modification sites, restrict their wide-scale application. Compared with their natural counterparts, artificial enzymes provide a variety of special benefits, including affordability, high stability, simplicity of modification, and versatile catalytic activity [[44], [45], [46], [47], [48]]. Nano-nuclease with properly designed multinuclear catalytic sites has been used for efficient cleavage of dsDNA [49]. There have been reports of numerous hydrolytic catalysts, often based on the complexes of Cu (II), Fe (III), Zn (II), Co (III), and Ce (IV), with the last being frequently the most active [39].

In recent years, nano platform-based combination therapy has become a brand-new avenue for the prospective treatment of inflammatory disorders [[50], [51], [52]]. Encouraged by these findings, we combined the strategies of DNA hydrolysis and STING suppression for stroke therapy. It is essential to halt the inflammatory signaling cascade through the degradation of immunogenic dsDNA stimulators and longer-acting specific inhibition of STING during the acute phase since pathogenic situations would cause STING in microglia to be hyperactive. It was reported that C-176 strongly reduced STING-mediated autoinflammatory processes [26].

Herein, a Ce^4+^ ligand-containing amphiphilic polymer, poly[caprolactone_*35*_-*b*-(oligoethylene glycol monomethyl ether methacrylate_*20.7*_-*co*-2,2'-((1-carboxy-5-methacrylamidopentyl)azanediyl) diacetic acid_*14*_)] (P[CL_*35*_-*b*-(OEGMA_*20*_-*co*-NTAMA_*14.3*_)]), was synthesized and self-assembled with C-176 to be a nanometer-scaled drug reservoir. Moreover, the NTA group was further coordinated with Ce^4+^ to achieve the corresponding drug-loading nano-nuclease (Fig. 2A). It was made up of three crucial parts: (i) Ce^4+^ coordinated with NTA to mimic DNase hydrolyzing immunogenic cell-free dsDNA from dead nerve cells. (ii) C-176, which was loaded in the hydrophobic PCL core and progressively released in physiological conditions, synergistically inhibited hyperactivated STING in microglia. (iii) Oligo ethylene glycol monomethyl ether methacrylate (OEGMA) was added to copolymerize with NTA-containing monomers to improve the stability and biocompatibility of the nanoparticles. Our research led to the development of nano-drugs that synergistically combines the DNase-memetic Ce^4+^ enzyme and STING inhibitor to modulate CNS inflammatory signaling pathways and boost the therapeutic impact of stroke prognosis.

**Fig. 1:**
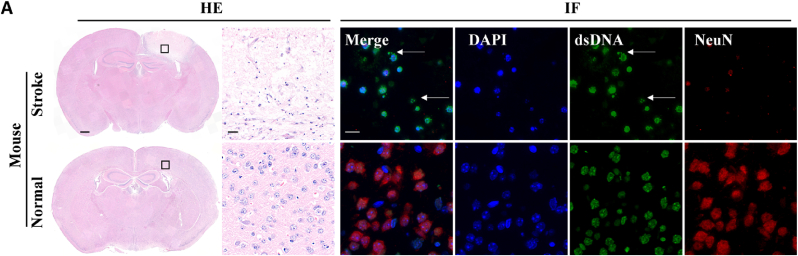
**The representative images of the HE staining, NeuN (red)/dsDNA (green) coimmunostaining of the MCAO model mice and corresponding controls.** Scale bars: 500 μm and 20 μm for HE staining and 20 μm for immunostaining. The dsDNA displayed cell-free dsDNA (green) as highlighted with white arrows.

**Fig. 2:**
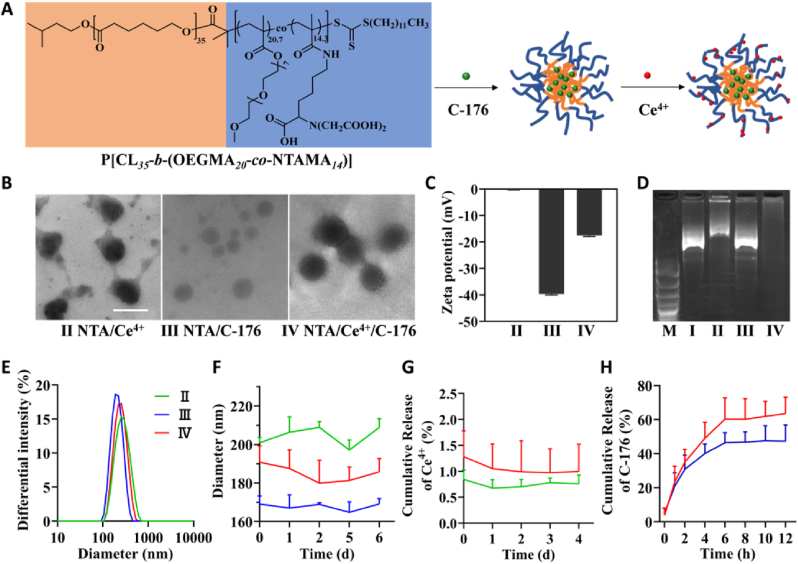
**The characterization of NTA/Ce**^**4+**^**/C-176 nanoparticles.** (A) Schematic illustration of amphophilic block copolymer P[CL_*35*_-*b*-(OEGMA_*20.7*_-*co*-NTAMA_*14.3*_)] structure and preparation of C-176-loaded nanoparticles coordinated with Ce^4+^. (B) TEM images of NTA/Ce^4+^, NTA/C-176, and NTA/Ce^4+^/C-176 freeze-dried in water; scale bar = 200 nm. (C) Zeta potential of NTA/Ce^4+^, NTA/C-176, NTA/Ce^4+^/C-176 in water (n = 3). (D) Agarose gel (1%) electrophoresis showing plasmids PUC18 (2686 bp) cleavage by Ce(NH_4_)_2_(NO_3_)_6_, NTA/Ce^4+^, NTA/C-176, NTA/Ce^4+^/C-176. Lane M: DNA marker; lane Ⅰ: PUC18 with Ce(NH_4_)_2_(NO_3_)_6_; lane Ⅱ: PUC18 with NTA/Ce^4+^; lane Ⅲ: PUC18 with NTA/C-176; lane Ⅳ: PUC18 with NTA/Ce^4+^/C-176. (E) The differential intensity of NTA/Ce^4+^, NTA/C-176, NTA/Ce^4+^/C-176. (F) Average hydrodynamic diameters of NTA/Ce^4+^, NTA/C-176, NTA/Ce^4+^/C-176 in water at 0, 1, 2, 5, 6 d (n = 3). (G) Cumulative release of Ce^4+^ from NTA/Ce^4+^ and NTA/Ce^4+^/C-176 at 0, 1, 2, 3, 4 d. The Ce signals were monitored by ICP-MS (n = 3). (H) Cumulative release of C-176 from NTA/C-176 and NTA/Ce^4+^/C-176 at 0, 1, 2, 4, 6, 8, 10, 12 h. The C-176 signals were monitored by HPLC (n = 6).

## 2 Methods

### 2.1 Synthesis of P[CL_*n*_-*b*-(OEGMA_*x*_-*co*-NTAMA_*y*_)]

The synthesis of (1*S*)–*N*-(5-amino-1-carboxypentyl)iminodiacetic acid (NTA-NH_2_), polycaprolactone macromolecular chain transfer agent (PCL_*n*_-CTA), and P[CL_*n*_-*b*-(OEGMA_*x*_-*co*-NHSMA_*y*_)] was detailed in supporting information [53,54]. For the synthesis of P[CL_*n*_-*b*-(OEGMA_*x*_-*co*-NTAMA_*y*_)]: P[CL_*n*_-*b*-(OEGMA_*x*_-*co*-NHSMA_*y*_)] (200.8 mg, 0.012 mmol; containing NHS 0.175 mmol, 1 eq.) was dissolved in DMSO (dimethyl sulfoxide) (1 mL) follow by addition of NTA-NH_2_ (68.6 mg, 0.262 mmol, 1.5 eq.) and *N*,*N*-diisopropylethylamine (25 μL). The solution was stirred at 50 °C for 48 h. The reaction mixture was diluted with DMSO and subsequently placed in clean dialysis tubes, dialyzed against ethanol and water many times. P[CL_*35*_-*b*-(OEGMA_*20.7*_-*co*-NTAMA_*14.3*_)] was finally obtained as a white powder (203.5 mg, 85% yield) after lyophilization. ^1^H NMR spectra were recorded on a Bruker DMX-400 MHz NMR and used to calculate the degree of polymerization.

### 2.2 Preparation of C-176 and Ce^4+^-loaded nano-zyme micelles (NTA/Ce^4+^/C-176)

The self-assembly formulation technique was employed to fabricate nanoparticles. Briefly, 5 mg of P[CL_*35*_-*b*-(OEGMA_*20.7*_-*co*-NTAMA_*14.3*_)] and 0.15 mg of C-176 were dissolved in 2.5 mL of dimethyl sulfoxide (DMSO), and the solution was dropwise added into 1.5 mL of intensely stirred water for 10 min. The mixture was dialyzed in purified water for 4 h and adjusted in volume to acquire 1 mg/mL NTA/C-176 water solution. For the preparation of Ce^4+^-loaded nanoparticles, 0.36 mg Ce(NH_4_)_2_(NO_3_)_6_ was added into 1 mL of 1 mg/mL NTA/C-176 solution with agitation and then dialyzed for 4 h (0.8 equivalent Ce^4+^ of NTA ligand has been demonstrated to be the optimized ratio to break the phosphodiester linkage [53].). The zeta potential and hydrodynamic diameter were assessed by dynamic light scattering (DLS, Zetasizer 3000, Malvern, USA) and morphology with the transmission electron microscope (TEM, HT-7700, Hitachi, Japan).

### 2.3 Stability and C-176 release of NTA/Ce^4+^/C-176

To test the stability of NTA/Ce^4+^/C-176 nanoparticles, the hydrodynamic diameters were detected by DLS at 0–6 d. 1 mg/mL NTA/Ce^4+^/C-176 nanoparticles were dialyzed in artificial cerebrospinal fluid (aCSF) at room temperature and the dialysis aCSF solution was collected to analyze the concentration of released cerium by inductively coupled plasma mass spectrometry (ICP-MS, Agilent 7850, USA). C-176 loading in NTA/Ce^4+^/C-176 was quantified using high-performance liquid chromatography (HPLC, Agilent 1260, USA) at 254 nm. The freeze-dried NTA/Ce^4+^/C-176 nanoparticles were suspended in a dialysis bag and stirred in purified water containing 0.1% Tween-20 at 37 °C. The dialysate was taken after 0, 1, 2, 4, 6, 8, 10, and 12 h. The mobile phase comprised acetonitrile: water (0.01% trifluoroacetic acid) = 54: 46 (v/v). The flow rate was 0.7 mL/min and the injection volume was 20 μL.

### 2.4 Cleavage of nucleic acids

0.2 mg/mL PUC18 plasmid solution was co-incubated with an equal volume of 0.36 mg/mL Ce(NH_4_)_2_(NO_3_)_6_, 1 mg/mL NTA/Ce^4+^, 1 mg/mL NTA/C-176 and 1 mg/mL NTA/Ce^4+^/C-176 (0.36 mg/mL Ce(NH_4_)_2_(NO_3_)_6_) solution at 37 °C for 8 h. The catalytic products were detected by agarose gel electrophoresis and gel red staining.

### 2.5 Biocompatibility and catalytic activity of NTA/Ce^4+^/C-176 *in vitro*

BV2 cells were grown in DMEM (Dulbecco's modified eagle medium) with 10% (v/v) FBS (Fetal Bovine Serum) (heat-inactivated at 56 °C for 30 min) and 1% (v/v) penicillin/streptomycin at 37 °C in an incubator with 5% CO_2_. CCK-8 (Cell Counting Kit-8) was used to assess the cytotoxicity of NTA/Ce^4+^/C-176 against BV2 cells. The cellular uptake was processed by co-incubating BV2 cells and 20 μg/mL NTA/Ce^4+^/Nile Red nanoparticles for 60 min. Flow cytometer (FACS caliber, BD Biosciences, USA) and confocal microscopy (Leica TCS SPE, Leica, Germany) were used to detect the fluorescence intensity of Nile Red in BV2 cells at 5, 30, and 60 min. Picogreen was applied to stain the cell-free dsDNA for the cleavage capability of NTA/Ce^4+^/C-176. BV2 microglia were seeded in a 12-well plate with DMEM and 10% FBS for 12 h. Then the medium was withdrawn, and DMEM with no glucose was used to wash the cells twice. The cells were then incubated in DMEM without glucose for 4.5 h at 37 °C in a 95% N_2_ and 5% CO_2_ environment to acquire the oxygen-glucose deprivation (OGD) model. Saline, 20 μg/mL NTA/Ce^4+^, NTA/C-176, and NTA/Ce^4+^/C-176 (0.36 mg/mL Ce(NH_4_)_2_(NO_3_)_6_) solution were administrated and incubated with microglia for 4 h at 37 °C in a 5% CO_2_ environment. After co-incubation, the supernatant was collected in black MicroWell with a transparent bottom and stained with pico-green detected under the fluorescence intensity Ex/Em = 480/520 nm by a microplate reader (Infinite M200, TECAN, Switzerland). Confocal pictures were obtained by seeding BV2 cells in 35 mm confocal plates and treated with what was previously stated. Instead of collecting supernatant, the cells were fixed with 4% paraformaldehyde solution, permeabilized with 0.5% Triton X-100, and blocked with 5% bovine serum albumin (BSA). The cells were incubated with the dsDNA marker and the cGAS antibody overnight at 4 °C, washed with PBS containing tween 20 (PBST), and incubated with secondary antibodies at room temperature for an hour. Then after PBST washing, the cells were stained with DAPI (2- (4-Amidinophenyl)-6-indolecarbamidine dihydrochloride), observed by confocal microscopy (Leica TCS SPE, Leica, Germany), and analyzed using LAS-AF-Lite software.

### 2.6 Western blot

Cells were lysed in RIPA buffer and separated by SDS-PAGE. Incubation with antibodies was performed in accordance to the manufacturing instructions, including pTBK1 (5483, CST), TBK1 (3013, CST), STING (13647s, CST), β-actin (4970, CST), and secondary antibody (2055718, Abcam). The expression levels of the target proteins were normalized to β-actin by Image J (Ver 1.52, NIH, USA).

### 2.7 Middle cerebral artery occlusion (MCAO) stroke model

The standards of all surgical procedures and subsequent care followed the guidelines of Animal Care and Use, Zhejiang Academy of Medical Sciences (Approval No. ZJCLA-IACUC-20020012). Eight-week-old male C57BL/6 mice weighing approximately 22 g were kept in a 12 h light/dark cycle with unrestricted access to food and water. Five groups of mice were randomly assigned: (1) saline, (2) 2 mg/mL edaravone + 0.5 mg/mL dexborneol, (3) 0.4 mg/mL NTA/Ce^4+^, (4) 0.4 mg/mL NTA/C-176, (5) 0.4 mg/mL NTA/Ce^4+^/C-176. The MCAO stroke model was induced by occluding the middle cerebral artery (MCA) by photochemical method following a reported procedure [9]. Briefly, using a gas anesthetic mask in a stereotaxic frame, mice were put to sleep with 2% isoflurane and kept that way with 1% isoflurane in an oxygen/air mixture. 30 mg/kg of 1% (w/w) Rose Bengal was administered intravenously 5 min before lighting. The skulls were made visible by shaving the scalp hairs and cutting up the scalps. Using H_2_O_2_, the periosteum over the skull was removed. The right sensorimotor cortex underwent irradiation to create an ischemic lesion. Group (1) and (3)–(5) received an intracerebroventricular injection at M/L: +1.0 mm, A/P: +0.5 mm of nanoparticles, and group (2) was given an intravenous injection of edaravone dexborneol after thrombosis. 2 μL of nanomaterials in total were injected over the course of 10 min at a rate of 0.2 μL/min and 100 μL of edaravone dexborneol were injected in 10 min at a rate of 10 μL/min. Using a micro-syringe with a 31 G needle for intracerebroventricular injection, it was taken out, waiting 5 min after administration. Mice were placed on a warm blanket after surgery until they were awake.

### 2.8 Cerebral infarct volume evaluation

The brains of mice (n = 4 for each group) were collected after 7 d and stained with 2% triphenyl tetrazolium chloride (TTC) after being sliced into 7 pieces sectioned coronally in stainless steel brain matrices. The white unstained cerebral infarct areas were measured by Image J.

### 2.9 Behavioral assessment

After therapy, the mice (n = 5 for each group) were subjected to behavioral tests on d 1, 3, and 7. The foot fault test was conducted at 1, 3, and 7 d. Mice were confined to a hanging steel grid with 1.2 cm by 1.2 cm perforations. The actions of mice were captured on camera for 5 min without disturbance. It was deemed an individual foot error when the injured paw (contralateral to infract hemisphere) completely missed the grid. The number of foot faults in the first 100 steps of the video served as the data. Another sensitive analysis to forecast the loss of forelimb motor function due to ischemic damage is the cylinder. A 500 mL transparent beaker with a layer of sawdust inside was used to place mice. Mirrors were positioned at an angle of 120° behind the cylinder so that the mouse's forelimb motion could be captured when it turned its back on the camera. The criteria for calculating the ratings for cylinder behavior were taken from previous reports [55]. On the seventh post-treatment d, a video was taken until the mouse made at least 20 movements. The final score is calculated as follows: (nonimpaired forelimb movement - impaired forelimb movement)/(nonimpaired forelimb movement + impaired forelimb movement + both movements). The lower the score, the better motor function is. The Zea-longa score was calculated using a recognized five-point scale [56] on the seventh d after treatment: 0 denoted no neurological deficiency, 1 denoted mild deficits (failure to extend the left forepaw completely), 2 denoted leftward circling, 3 denoted leftward falling, indicative of moderate deficits, and 4 denoted severe deficits (the complete loss of walking ability). Mice with scores between 1 and 4, which indicated successful MCAO modeling, were involved in the following studies. In the behavior assessment, the researchers were blind to the statistics of behavioral parameters.

### 2.10 Histology and immunofluorescence analysis

The brains of mice (n = 3) were collected after 7 d and were fixed in 4% paraformaldehyde for 24 h. Then they were sliced in stainless steel brain matrices respectively, dehydrated in graded ethanol, embedded in paraffin, and cut into 5 μm-thick slices for immunofluorescence analysis. Using 5% BSA, brain slices were blocked for 1 h at room temperature. Thereafter, brain slices were then incubated at 4 °C overnight with the listed below primary antibodies: ds DNA Marker (HYB331-01): sc-58749 (1:100, Santa Cruz Biotechnology); Anti-NeuN antibody (ab104225) (1:100, Abcam); STING (D2P2F) (#13647) (1:100, Cell Signaling Technology); and Recombinant Anti-Iba1 antibody (ab178847) (1:100, Abcam). Following a PBST wash, slices were incubated for 2 h at room temperature with the corresponding secondary antibodies with fluorescent labels. The obtained slices were washed before their nuclei were stained with DAPI for 5 min. In the end, CaseViewer was used to evaluate the images detected by the slide scanner (Axio Scan. Z1, Zeiss, Germany).

### 2.11 Biosafety *in vivo*

Three groups of mice were settled: Group (1) is healthy without any medication; Group (2) is an intracerebroventricular injection (ICV) with 2 μL of 0.4 mg/mL NTA/Ce^4+^/C-176; Group (3) is an intravenous injection (IV) with 100 μL of 0.4 mg/mL NTA/Ce^4+^/C-176. *In vivo* toxicity, including blood routine, blood biochemical, and histopathology assays, were examined on 7 d with the following medication. Hematoxylin-eosin (H&E) staining was used to examine the heart, liver, spleen, lung, kidney, and brain in sections. Blood was collected, and the levels of alanine aminotransferase (ALT), aspartate aminotransferase (AST), blood urea nitrogen (BUN), creatinine (CRE), red blood cell count (RBC), platelet count (PLT) neutrophil count (Neu) lymphocyte count (Lym) were promptly determined.

### 2.12 Statistical analysis

The mean and standard error of the mean (SEM) were used to express experimental data. The one-way ANOVA with Tukey's multiple comparison tests in GraphPad was used to examine the significant differences between groups. Statistics were deemed significant at *P* < 0.05.

## 3 Results and discussion

### 3.1 Accumulation of dsDNA and loss of neurons in ischemic stroke

The role of dsDNA as an inflammatory driver in stroke was initially investigated. As shown in Fig. 1A (HE staining), the ischemic area appeared whiter because it could not bind to eosin. The expanded images showed numerous clustered cells in the infarct area were accompanied by fragmentation of free extracellular dsDNA in blue. These fragments of dsDNA did not have the distinctive morphology of the nucleus and were scattered between dead nerve cells, which are our therapeutic targets. More specifically, the immunofluorescence labeling revealed that the neuronal functions were lost in ischemic tissue, as reflected by the downregulated fluorescence of red NeuN, a characteristic of the neurons. The nuclear-form dsDNA of dead cells was densely stained in green and continuously leaked out fragmented dsDNA into the microenvironment as an inflammatory signal, suggesting profound dsDNA-related aseptic immune inflammation.

### 3.2 Characterization of P[CL_*n*_-*b*-(OEGMA_*x*_-*co*-NTAMA_*y*_)]

NTA-NH_2_ and PCL_*n*_-CTA were synthesized first, and ^1^H NMR and GPC (Gel Permeation Chromatography) were employed to characterize their chemical structures, as shown in Figs. S1–S5. The number of structural units of P[CL_*n*_-*b*-(OEGMA_*x*_-*co*-NHSMA_*y*_)] was calculated as *n* = 35, *x* = 20.7, *y* = 14.3, based on the ^1^H NMR results (Fig. S6). It can be seen in Fig. S7 that the chemical shift of NHS hydrogen has almost disappeared, indicating that NHS has completely reacted with NTA-NH_2_.

### 3.3 Characterization of NTA/Ce^4+^/C-176

**Fig. 3:**
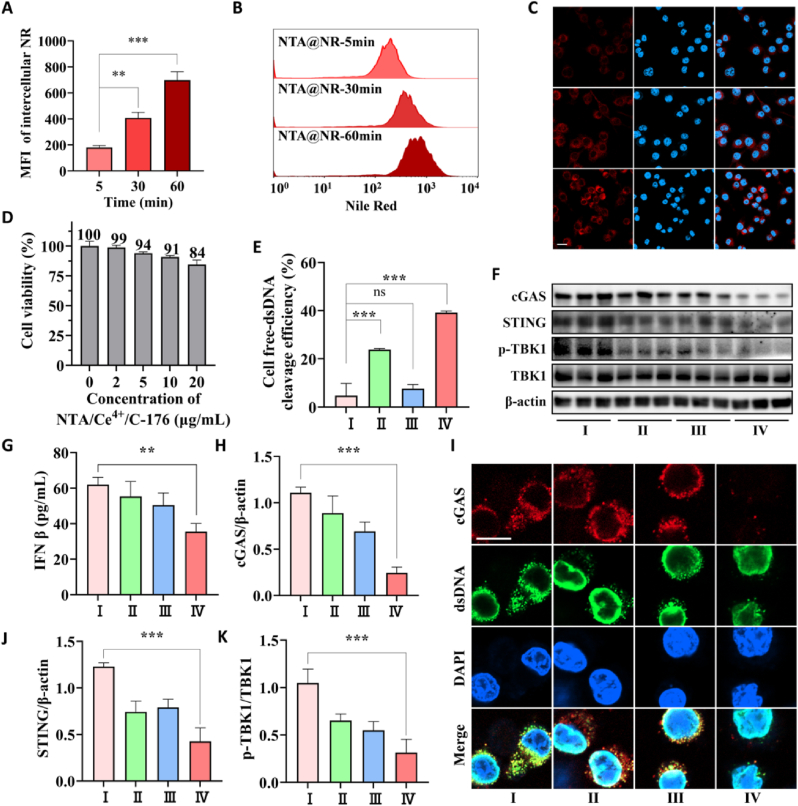
**Biocompatibility and catalytic activity of NTA/Ce**^**4+**^**/C-176 *in vitro*.** (A) Quantified NTA/Ce^4+^/Nile red cellular uptake in BV2 cells by flow cytometry analysis with increasing co-incubation time (n = 3). Data are presented as mean ± SD. **P < 0.01, ***P < 0.001. (B) Typical flow cytometry data of cellular uptake of NTA/Ce^4+^/Nile red in BV2 cells. (C) Confocal images of intracellular NTA/Ce^4+^/Nile red in BV2 cells. NTA/Ce^4+^/Nile red indicated by a red signal and nuclei stained by DAPI indicated by a blue signal; scale bar = 20 μm. (D) Viability of BV2 cells treated for 24 h with various concentrations of NTA/Ce^4+^/C-176 (n = 3). (E) Cell-free dsDNA cleavage efficiency of the pico-green-stained BV2-cultured supernatant. Data are means ± SEM (n = 3 independent experiments; ***P < 0.001 by one-way ANOVA with Tukey's multiple comparison tests, ns means no significant difference). (F) Representative immunoblotting of cGAS, STING, p-TBK1, and TBK1 in BV2 after indicated treatments (n = 3), using β-Actin as control. ELISA of (G) IFNβ, blotting quantification of (H) cGAS, (J) STING, and (K) p-TBK1. (I) Cellular immunofluorescence images of dsDNA (green) and cGAS (red) in BV2 cells. Scar bar = 10 μm.

Amphipathic P[CL_*35*_-*b*-(OEGMA_*20.7*_-*co*-NTAMA_*14.3*_)] self-assembled in water by a hydrophilic random copolymer of P(OEGMA_*20.7*_-*co*-NTAMA_*14.3*_) as a shell and a hydrophobic block of PCL_*35*_ as a core (Fig. 2A). C-176 is a small-molecule inhibitor with poor solubility and could be encapsulated into the hydrophobic inner core by a self-assembled hydrophobic interaction force. Ce^4+^, which binds to the carboxyl groups of NTA by coordination bonds, was reported [53] to form active catalytic centers as DNase mimetics for catalyzing the hydrolysis of DNA. The diameters of Ce^4+^ coordinated NTA nanoparticles (NTA/Ce^4+^), C-176 loaded NTA nanoparticles (NTA/C-176), and C-176 loaded NTA nanoparticles coordinated with Ce^4+^ (NTA/Ce^4+^/C-176) (Fig. 2B, E and F) were measured at around 200.9 nm (PDI: Polymer dispersity index = 0.15), 169.1 nm (PDI = 0.06) and 190.8 nm (PDI = 0.10). The corresponding zeta potential was around 0 mV, −40 mV, and −18 mV in water (Fig. 2C), respectively. As reported, a large density of cationic modification may indiscriminately adsorb negatively charged biomacromolecules resulting in nonspecific toxicity [32]. What's worse, the accumulation of dsDNA leads to the saturation of adsorption sites. In this case, the negatively charged Ce DNase (NTA/Ce^4+^/C-176) might exhibit superior biocompatibility (Fig. 3D). As shown in (Fig. 2F), the PEGylation led to a 7-d dispersion stability in water. Moreover, we investigated the stability of Ce^4+^ coordinated with NTA in a simulated physiological environment by immersing the NTA/Ce^4+^ and NTA/Ce^4+^/C-176 nanoparticles in the artificial cerebral spinal fluid (aCSF) for 4 d. There was only 0–1.0% of cerium (Fig. 2G) released according to the detection results of ICP-MS, indicating good stability of the catalytic structures.

Since STING would be overactivated in microglia under pathological conditions, it is significant to inhibit the inflammatory effector during the acute phase. Small molecules have insufficient retention times in the brain. Thus, a nanometer-scaled drug reservoir could be a preferred dosage form to acquire a long-term inhibition of STING hyperactivation by C-176 sustained release. The NTA/Ce^4+^, NTA/C-176, and NTA/Ce^4+^/C-176 were constructed with a C-176 loading efficiency of 0%, 1.9%, 1.4%, and Ce^4+^ loading efficiency of 94%, 0%, 71%, respectively. C-176 was tightly encapsulated in the hydrophobic inner core and was continuously released from nanoparticles within 12 h (Fig. 2H) in a stirring water solution containing 0.1% Tween-20 at 37 °C.

Moreover, the complexes of Ce^4+^ and NTA could be a supplement to the brisk demand for nuclease in the ischemic area [12]. We next investigated the dsDNA hydrolytic activity of the Ce enzymes with the same equivalent Ce^4+^ concentration (Fig. 2D). We found that plasmids PUC18 (2686 bp) were cleaved into small fragments, as seen in 2% agarose gel electrophoresis stained with gel red in group NTA/Ce^4+^ and NTA/Ce^4+^/C-176. In contrast, PUC18 treated with Ce(NH_4_)_2_(NO_3_)_6_ stoke solution and NTA/C-176 showed limited cleavage activity with brighter bands. These results confirmed the notion that the Ce^4+^ active sites were the unambiguous source of the DNase-mimetic function of NTA/Ce^4+^ and NTA/Ce^4+^/C-176 while there were no stable catalytic activities in Ce(NH_4_)_2_(NO_3_)_6_ stoke solution alone. It is reported that the phosphate's electrons in phosphodiester linkage are strongly withdrawn by the two adjacent Ce^4+^ centers and were activated by the nucleophilic attack of hydroxide ions, resulting in the hydrolysis of phosphodiester bonds in DNA [57]. Especially, the clear lane Ⅳ indicated the complete degradation of the long chain plasmids by NTA/Ce^4+^/C-176. The hydrolysis was facilitated by the more stable assembly structure of NTA/Ce^4+^/C-176 to offer more efficient and stable catalytic sites, which could be utilized as a universal nano-nuclease to replace the natural counterparts.

### 3.4 Biocompatibility and catalytic activity of NTA/Ce^4+^/C-176 *in vitro*

The murine-microglia-derived BV2 cell lines, which are extensively employed in the research of neurophysiology and neuropharmacology, have typical morphological and functional features of primary microglia. As a result, BV2 cells were selected as the model cells for the following study. To address the safety issues with NTA/Ce^4+^/C-176, an *in vitro* evaluation was initially carried out since biocompatibility is essential for the deployment of nanomaterials. After 24 h of co-incubation with NTA/Ce^4+^/C-176 at a concentration of less than 20 μg/mL, the viability of BV2 cells as determined by the CCK8 assay exceeded 85% (Fig. 3D). Due to the negatively charged surface of nanoparticles, there was no toxicity from cationic adsorption of biological macromolecules, improving the biological safety of NTA/Ce^4+^/C-176. Moreover, as shown in Fig. 3A–C, particles could be engulfed by BV2 cells within 60 min, suggesting that the biocompatible negative surface did not affect the endocytosis of particles in microglia.

The neuroinflammation *in vitro* was simplified to the oxygen-glucose deprivation (OGD) model. In the sterile inflammation that follows an ischemic stroke, dsDNA is a powerful DAMP [23] released into the extracellular environment and engulfed by microglia. To investigate the catalytic capability of nanoparticles, OGD-BV2 cells were incubated with saline, 20 μg/mL NTA/Ce^4+^, NTA/C-176, and NTA/Ce^4+^/C-176 solution, which was the safe concentration tested by CCK8 previously.

The levels of dsDNA were stained using a dsDNA marker with no nuclear membrane permeability and detected by confocal microscopy. As illustrated in Fig. 3I, BV2 cells treated with saline and NTA/C-176 gobbled up large amounts of dsDNA. Free green fluorescent debris permeated the cytoplasm and intercellular microenvironment. In contrast, group NTA/Ce^4+^ and group NTA/Ce^4+^/C-176 kept the accumulation of dsDNA lower. Additionally, pico-green assay was used to measure the amounts of dsDNA in the cell culture supernatant, and the saline-treated group was set as the control. As shown in Fig. 3E, dsDNA accumulation was noticeably reduced after 12 h of incubation with NTA/Ce^4+^/C-176. The efficiency of cell free-dsDNA cleavage in the OGD cell model using NTA/Ce^4+^ and NTA/Ce^4+^/C-176 was around 23.8% and 39.2%, respectively. The experimental outcomes supported nanoparticles' capacity to accelerate the degradation of dsDNA. In group NTA/Ce^4+^/C-176, C-176 released by nanoparticles could synergistically suppress STING hyperactivation downstream of the innate signaling pathway and lessen inflammatory factors secreted into the neuro-microenvironment. As a result, less free DNA was produced by inflammation-damaged cells, effectively defending nerve cells against inflammatory assaults. It suggested that C-176 and Ce enzymes worked together to scavenge dsDNA by interrupting the negative inflammatory feedback. Even more, to see how dsDNA and cGAS were localized together, we co-stained cGAS [16] in red (Fig. 3I). The cGAS-STING axis remained active, as shown in Fig. 3 G, H, J K, and I after DNA clearance or STING inhibition alone. The cGAS-DNA complexes can only be completely removed if Ce DNase and STING inhibitors are used in tandem, also indicating a synergistic mechanism of C-176 and Ce enzymes in dsDNA scavenging, and it would be reinforced *in vivo* assays.

### 3.5 Effect of NTA/Ce^4+^/C-176 on brain infarct volume and functional motor recovery after stroke

**Fig. 4:**
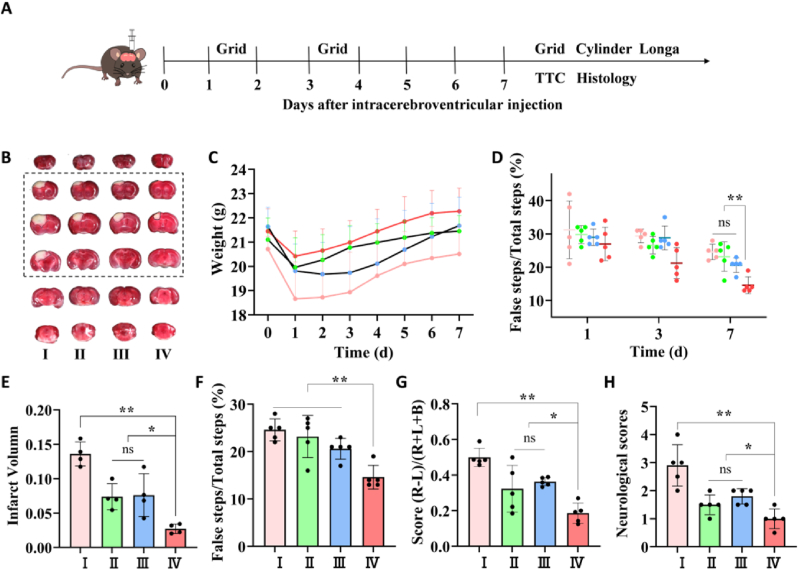
**Effect of NTA/Ce**^**4+**^**/C-176 on brain infarct volume and functional motor recovery after stroke.** (A) The experimental timeline. (B) Representative images of the TTC-stained brain slice after 7 d post-stroke. The normal tissue was stained red, while the injured tissue was unstained white. (C) Body weight before cerebral infarction (d 0) and after cerebral infarction with different treatments (n = 5). (D) Grid test on 1, 3, and 7 d after stroke. (n = 5). (E) Infarct volume of the TTC-staining at 7 d post-stroke (n = 4). (F) Grid test on 7 d after stroke (n = 5). (G) Cylinder test on 7 d after therapy (n = 5). (H) Neurological scores on 3 d after therapy (n = 5). *P < 0.05, **P < 0.01, ns means no significant difference.

The photochemically generated cerebral infarction was employed for the middle cerebral artery occlusion (MCAO) mode, which is usually applied to examine possible stroke neuroprotective treatments. Edaravone dexborneol, a new neuroprotective drug with synergistic antioxidant and anti-inflammatory actions, was demonstrated to increase the proportion of patients with favorable functional results 90 d after randomization in a phase III trial [58]. Here we used it as a positive control. After being randomly assigned to one of five groups, stroke mice were immediately intracerebrally injected with (1) saline, (3) 0.4 mg/mL NTA/Ce^4+^, (4) 0.4 mg/mL NTA/C-176, (5) 0.4 mg/mL NTA/Ce^4+^/C-176 and intravenously injected with (2) 2 mg/mL edaravone + 0.5 mg/mL dexborneol. Data of the Edaravone group will be presented in Figs. S8–S9. During the initial days following an injury, mice frequently lost body weight (Fig. 4C). 1, 3, and 7 d after various treatments, behavioral assessments in response to ischemia damage were evaluated, and the infarct volumes and pathological sections were analyzed 7 d after therapy (Fig. 4B and S8). The cerebral infarct volume was quantified by triphenyl tetrazolium chloride (TTC) staining, displaying the stained normal tissue in red and the unstained infraction in white. The infraction regions (white) of the saline and edaravone dexborneol groups reached 13.6% and 11.7% of the entire brain sections, respectively, according to Fig. 4E and S8, whereas they were 7.4%, 7.6%, and 2.7% in the NTA/Ce^4+^ group, NTA/C-176 group, and NTA/Ce^4+^/C-176 group, respectively, suggesting that when compared to an equivalent dosage of nanoparticles, NTA/Ce^4+^/C-176 exhibited superior protective effectiveness.

Stroke also results in impairments in limb usage. Therefore, three behavioral methods were adopted to assess the motor recovery of mice for 1, 3, and 7 d. The dexterity and flexibility of the contralateral forelimb were evaluated by the grid test, cylinder test, and Zea-longa score test (Fig. 4F, G, and H and S8), respectively. In the cylinder test, the forelimb usage of the stroke mice was markedly lopsided, with a propensity to contact or support the cylinder wall with the unaffected forelimb. In the grid test, the stroke mice were more likely to step outside the grid. Since edaravone was administered intravenously, its therapeutic efficacy was constrained by poor blood-brain barrier permeability and limited curative time. The NTA/Ce^4+^, NTA/C-176, and NTA/Ce^4+^/C-176 groups intracerebroventricular injected showed more statistically effective results in the acute period with less body weight loss and more recovery of limb coordination (Fig. 4C, D and S8). Furthermore, the mice treated with the NTA/Ce^4+^/C-176 showed the most considerable decrease in the neurological score (Fig. 4H and S8), the asymmetric touches (Fig. 4G and S8), and the miss steps (Fig. 4F and S8) after 7 d post-injury. It was the combination of Ce^4+^ nano-nuclease and C-176 that enhanced therapeutic effectiveness compared with the groups of ingredients alone, which was capable of substantially lessening ischemic penumbra and promoting functional motor recovery.

### 3.6 NTA/Ce^4+^/C-176 decreased neuroinflammation and increased neurogenesis *in vivo*

**Fig. 5:**
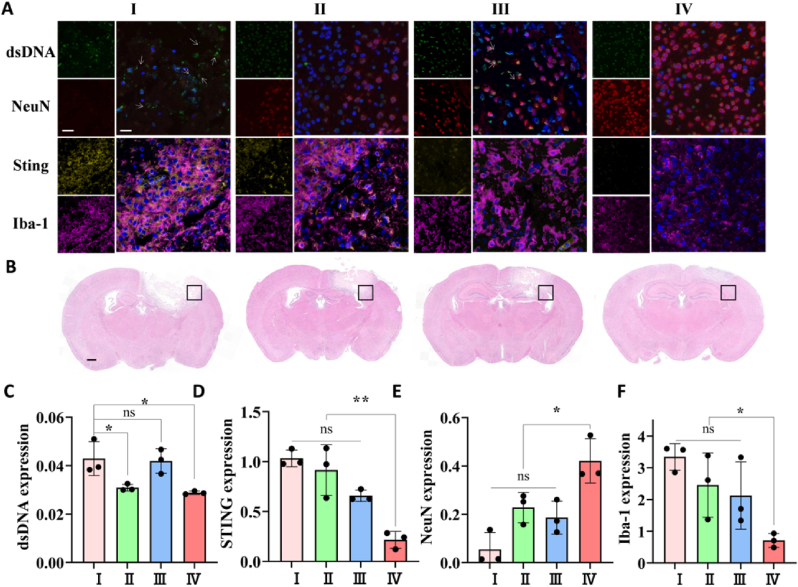
**NTA/Ce**^**4+**^**/C-176 decreased neuroinflammation and increased neurogenesis *in vivo*.** (A) Immunofluorescence of dsDNA (injury markers, green), NeuN (neuron markers, red), DAPI (nucleus markers, blue), STING (injury markers, yellow), Iba-1 (microglia markers, purple). The white arrows indicated dsDNA-positive area (injury); scale bar = 20, 40 μm). (B) The corresponding representative immunohistochemical brain slices of the immunofluorescence pictures (scale bar = 500 μm). (C) Quantitative analysis of relative dsDNA levels (n = 3), STING levels in (D) (n = 4). NeuN levels in (E) and Iba-1 in (F) (n = 3) normalized to nuclei, respectively. *P < 0.05, **P < 0.01, ns means no significant difference.

A vital indicator of the apoptosis of susceptible neurons is dsDNA, which reflects the degree of ischemic injury and induces an innate immune cascade in microglia. The white arrows point to the green fluorescent fragments indicating the accumulated dsDNA from apoptotic nerve cells (Fig. 5A, B, C and S9). As shown in the immunofluorescent images, there was essentially no NeuN red staining of neurons but with free fragments of dsDNA and cell debris in the amplified region of saline and edaravone groups. The lesion was not effectively treated over the 7-d treatment period when free radical scavengers edaravone were injected peripherally. In contrast, the NTA/Ce^4+^ and NTA/Ce^4+^/C-176 groups were found an obvious clearance of green fluorescent fragments, and the majority of the dsDNA green staining was colocalized with DAPI blue and NeuN red, indicating that the dsDNA was the intact genetic material in healthy neurons. These revealed how dsDNA segments hydrolyzed by cerium nano-nuclease contributed to the survival of neurons (Fig. 5C, E, S9 and 10) in the ischemic penumbra. Neuronal death and the development of subacute infarctions are both significantly influenced by microglia-based neuroinflammation [59]. There were also barely detectable NeuN signs of neuronal rescue in group NTA/C-176 but still excessive dsDNA surrounding the nucleus, suggesting that nano-sized C-176 medications are limited to clearing inflammatory DNA and do not aim at the etiology of inflammation. It was further proved in subsequent experiments that the incorporation of artificial nucleases into the system was an effective therapeutic to replace the pricey and unstable natural DNase in the microenvironment *in vivo*.

Aseptic neuroimmune inflammation refers to the recruitment and proliferation of microglia in the peri-infract areas (Fig. 5A, F, S9 and 10). Because microglia are in charge of the phagocytosis of the accumulating dsDNA, the cGAS-STING axis is mostly hyperactivated in these cells. STING (yellow) was overexpressed in proliferated microglia located at the margin of the ischemic damage (Fig. 5A, D and S9) in the saline and edaravone groups. Edaravone did not interfere with the specific immune pathways with limited curative effect. However, as compared to the saline group, the NTA/C-176 and NTA/Ce^4+^/C-176 NPs intracerebroventricularly injected into the stroke site produced an obvious decrease of 38.8% and 82.9% of the expression levels of STING (Fig. 5D and S9) in brain homogenate. Moreover, the consequent inhibition of microglia proliferation (Fig. 5F and S9 and 10) was also accomplished by specifically blocking the effects of the hyperactivated STING protein by C-176 and efficiently hydrolyzing dsDNA by Ce enzymes. Consistently, the western blot showed a slight downregulation of STING in the NTA/Ce^4+^ group of 19.7%, indicating that although the separate artificial enzyme contributes to attenuating the innate inflammatory cascade, only the combination of both components can significantly increase the effectiveness. STING, the effector of innate immune activation, the coordinated regulation of which reversed the acute inflammatory processes. Encapsulating C-176 into Ce enzymes, which enhanced retention time and systematic compatibility of small molecules, aids in the further application of the DNase-memetic nano-nuclease for stroke therapy. It is a novel attempt at the utilization of Ce-based nano-nucleases for neuroinflammatory diseases. Immunotherapies are promising strategies for immuno-dyshomeostasis followed by ischemia, and the constrained efficacy of individual bioactive agents could be overcome by nanoplatforms for broader implementation of neuroprotection.

### 3.7 Biocompatibility of NTA/Ce^4+^/C-176 *in vivo*

**Fig. 6:**
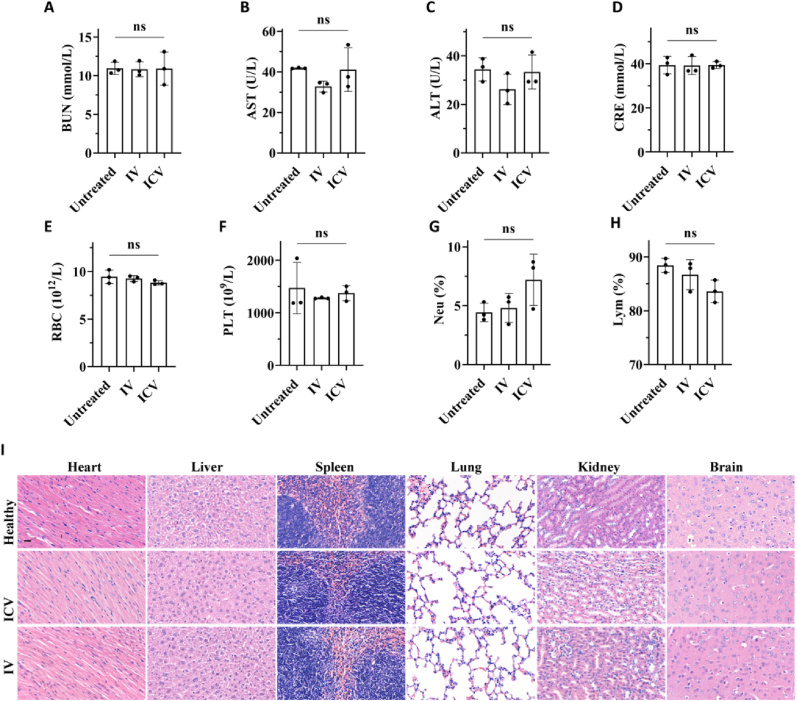
**Toxicological evaluations after 7 d of treatment via different injections.** (A–D) Blood biochemical values of blood urea nitrogen (BUN), aspartate aminotransferase (AST), alanine aminotransferase (ALT), and creatinine (CRE) at 7 d post-injection (n = 4). (E–H) Routine blood indexes of red blood cell (RBC), platelets (PLT), neutrophil (Neu), and lymphocytes (Lym) at 7 d post-injection. ns means no significant difference. (I) H&E staining of heart, liver, spleen, lung, kidney, and brain at 7 d post-injection; scar bar = 20 μm.

The biosafety of NTA/Ce^4+^/C-176 *in vivo* was quantified by blood biochemical values and blood routine indexes. As shown in Fig. 6A–D, the BUN, AST, ALT, and CRE did not alter across the various therapies, showing that the liver and kidney functions were not impeded. Fig. 6E–H shows fluctuations in the blood cell counts, but no significant difference was observed. By day 7 post-injection, no discernible abnormality was found on H&E staining pictures of the heart, liver, spleen, lung, kidney, and brain (Fig. 6I), indicating no serious harm to major organs. The outcomes confirmed NTA/Ce^4+^/C-176's biocompatibility and prospects as a secure option for the treatment of stroke.

## 4 Conclusions

The stroke-damaged neuroimmune microenvironment is an aseptic region with hyperactivated innate immune inflammation. It is directly associated with dsDNA accumulation and STING overexpression, which is the negative feedback of destroying neuroimmune homeostasis. Given that our study constructed a novel nano-drug combining DNase-memetic Ce^4+^ enzyme and STING inhibitor for balancing the cGAS-STING axis to improve the therapeutic effect of stroke. The synergistic combination of artificial nano-nuclease and C-176 was more effective than their individual bioactive components. This is because the combination of DNA clearance and targeted inhibition of STING can alleviate the inflammation caused by the activation of cGAS-STING innate immune pathways, thus saving the neurons in the ischemic penumbra from secondary inflammatory damage. The survival of more neurons in the damaged area can also reduce the apoptotic dsDNA content to help alleviate STING hyperactivation, therefore achieving the synergistic therapeutic effect based on positive feedback. This research demonstrated an innovative strategy for modulating CNS inflammatory signaling pathways and promoting stroke prognosis.

## CRediT authorship contribution statement

**Zhixin Zhu:** Conceptualization, Methodology, Validation, Formal analysis, Investigation, Writing – original draft. **Haipeng Lu:** Methodology, Validation, Formal analysis, Investigation, Writing – original draft. **Lulu Jin:** Methodology, Validation, Formal analysis. **Yong Gao:** Methodology. **Zhefeng Qian:** Methodology, Validation, Formal analysis. **Pan Lu:** Formal analysis. **Weijun Tong:** Methodology. **Pik Kwan Lo:** Methodology. **Zhengwei Mao:** Conceptualization, Methodology, Supervision, Writing – review & editing, Funding acquisition. **Haifei Shi:** Methodology.

## Declaration of competing interest

The authors affirm that they have no known financial or interpersonal conflicts that could have appeared to have an impact on the research presented in this study.
