# Supplementary material for: C-176 loaded Ce DNase nanoparticles synergistically inhibit the cGAS-STING pathway for ischemic stroke treatment
Source: Bioact Mater. 2023 Jul 18;29:230–40. doi: 10.1016/j.bioactmat.2023.07.002 (PMC10371767; doi:10.1016/j.bioactmat.2023.07.002)
Supplement: Multimedia component 1 [file mmc1.docx]

**Supplementary Information**

**C-176 loaded Ce DNase nanoparticles synergistically inhibit the cGAS-STING pathway for ischemic stroke treatment**

**C-176 loaded Ce DNase nanoparticles synergistically inhibit the cGAS-STING pathway for ischemic stroke treatment**

Zhixin Zhu^1,2#^, Haipeng Lu^2#^, Lulu Jin^2^, Yong Gao^2^, Zhefeng Qian^2^, Pan Lu^1^, Weijun Tong^2^*, Pik Kwan Lo^3^*, Zhengwei Mao^2^*, Haifei Shi^1^*

1. Department of Orthopedics, 1st Affiliated Hospital of Zhejiang University School of Medicine, Qingchun Road 79, Hangzhou, 31000, China

2. MOE Key Laboratory of Macromolecular Synthesis and Functionalization, Department of Polymer Science and Engineering, Zhejiang University, Hangzhou 310027, China

3. Department of Chemistry and State Key Laboratory of Marine Pollution, City University of Hong Kong, Tat Chee Avenue, Kowloon, Hong Kong

**#** These authors contribute equally.

*** Corresponding authors:**

Weijun Tong ([tongwj@zju.edu.cn](mailto:tongwj@zju.edu.cn))

Pik Kwan Lo (peggylo@cityu.edu.hk)

Zhengwei Mao (zwmao@zju.edu.cn)

Haifei Shi (shihaifei@zju.edu.cn)

**Chemicals**

Ammonium ceric nitrate (Ce(NH_4_)_2_(NO_3_)_6_, Aladdin, ≥ 99.99% metals basis), *ε*-caprolactone (*ε*-CL, Macklin, 99 %), 2-(dodecylsulfanylthiocarbonylsulfanyl)-2-methylpropionic acid (Macklin, 97 %), 1,5,7-triazabicyclo[4.4.0]dec-5-ene (TBD, Macklin, 97 %), oligoethylene glycol monomethyl ether methacrylate (Macklin, average molecular weight about 452 g/mol), *N*-succinimidyl methacrylate (Macklin, 98 %), α,α'-azoisobutyronitrile (AIBN, Macklin, 99 %), *N*-benzyloxycarbonyl-L-lysine (Macklin, 98 %), bromoacetic acid (Aladdin, 98 %), 10% Pd/C catalyst (Energy Chemical, wetted with ca. 55% Water), 3-methyl-1-butanol (Macklin, 99 %, with molecular sieves, water ≤ 50 ppm), *N*, *N*-diisopropylethylamine (Macklin, 99.5 %), benzoic acid (Macklin, 99 %), 4-dimethylaminopyridine (DMAP, Aladdin, 99%), 1-ethyl-3-(3-dimethylaminopropyl) carbodiimide hydrochloride (EDC·HCl, Macklin, 98 %), C-176 (MedChemExpress, 99.45 %). BV2 cell line (CL-0493) was purchased from Procell Life Science & Technology Co., Ltd. Dulbecco's modified Eagle's medium (DMEM), fetal bovine serum (FBS), and penicillin/streptomycin were purchased from Gibco. Cell counting kit-8 (CCK-8) was purchased from Target Molecule Corp. Plasmid PUC18, and picogreen was purchased from Solarbio. Anti-Iba1 antibody (ab283319), anti-NeuN antibody (ab177487), and goat polyclonal secondary antibodies to rabbit IgG-H&L (Alexa Fluor® 488, ab150077 and Alexa Fluor® 647, ab150079) were purchased from Abcam. STING (D2P2F) Rabbit mAb #13647 was purchased from Cell Signaling Technology. Ds DNA Marker Antibody (HYB331-01): sc-58749 was purchased from Santa Cruz Biotechnology. 2,3,5-triphenyl tetrazolium chloride (TTC) was purchased from Shanghai Yuanye Bio-Technology Co., Ltd. Unless otherwise stated, organic solvents and inorganic salts were obtained from Sinopharm Chemical Reagent Co., Ltd. and utilized without additional purification. Water used in all the experiments was purified by the Millipore-Q water-purification System (Bedford, USA). Molecular weights and distributions were measured by gel permeation chromatography (GPC) on a Waters 1515 system at 40°C. Tetrahydrofuran (THF) was used as the eluent with a 1.0 mL/min flow rate, and the mono-dispersed polystyrenes were used as standards. ^1^H NMR spectra were recorded on a Bruker DMX-400 MHz NMR and used to calculate the degree of polymerization.

**Synthesis of NTA-NH_2_** [1]

(i) *N*-benzyloxycarbonyl-L-lysine (8.41 g, 30 mmol, 1 eq.) was dissolved in 45 mL of 2 M NaOH, and the solution was added dropwise (~10 min) with stirring to a cooled solution (0 °C) of bromoacetic acid (8.34 g, 60 mmol, 2 eq.) in 2 M NaOH (30 mL). The solution was stirred overnight at 25°C, and after heating for 2 h at 70 °C, 1 M HCl (90 mL) was added to the cooled solution. The precipitate was filtered off and dried to afford a crude white powder, purified by further dissolution in 1 M NaOH (100 mL) and precipitation with 1 M HCl (100 mL) to give pure (*1S*)-*N*-(5-carbobenzyloxyamino-1-carboxypentyl)iminodiacetic acid (compound 1, 10.58 g, yield 89.0 %). The ^1^H NMR spectrum (400 MHz, DMSO-*d*_6_) is shown in Figure S1.

(ii) A solution of compound 1 (6.83 g, 17.2 mmol) in methanol (108 mL)/H_2_O (5.7 mL) and 10% Pd/C catalyst (0.68 g) was stirred in H_2_ at 25 °C and 760 mmHg overnight. The solution was filtered, and the filter residue was dispersed in Water (50 mL). Then, the suspension was filtered, and a white powder was obtained after lyophilization of the filtrate. The powder was redissolved in H_2_O (20 mL), and then ethanol (15 mL) was added until the solution became cloudy; after heating to give a limpid solution, the mixture was allowed to stand at -20 °C with seeds. The white crystals were filtered off and dried to afford (*1S*)-*N*-(5-amino-1-carboxypentyl)iminodiacetic acid (NTA-NH_2_, 4.37 g, yield 96.7 %). The ^1^H NMR spectrum (400 MHz, D_2_O) is shown in Figure S2.

**Synthesis of PCL*_n_*-CTA**

(i) PCL-OH was synthesized according to the literature [2]. In a 100 mL flame-dried Schlenk flask equipped with a stirring bar, *ε*-CL(11.30 g, 100 mmol, 20 eq.) was added to a solution of TBD (70 mg, 500 μmol, 0.1 eq.) and 3-Methyl-1-butanol(0.55 mL, 0.44 g, 5 mmol, 1 eq.) in toluene (50 mL). The solution was stirred for 5.5 h and quenched by adding benzoic acid. Then, it was concentrated under a vacuum and precipitated in diethyl ether to give the product a white powder (9.27 g, yield 82%). The ^1^H NMR spectrum (400 MHz, CDCl_3_) is shown in Figure S3. The molecular weight and molecular weight distribution(MWD) of PCL-OH were determined by gel permeation chromatography (GPC) using tetrahydrofuran(THF) as the eluent, revealing a number-average molecular weight of 8.2 kDa and MWD=1.13 (Figure S4). The actual degree of polymerization (DP) of the PCL-OH was determined to be 35 by ^1^H NMR analysis.

(ii) PCL-OH (8.01 g, 1.96 mmol, 1 eq.), 2-(dodecylsulfanylthiocarbonylsulfanyl)-2-methyl propionic acid (1.43 g, 3.92 mmol, 2 eq.) and DMAP (239 mg, 1.96 mmol, 1 eq.) were dissolved in 40 mL of dichloromethane (DCM) and cooled in an ice bath. Then, EDC HCl (1.50 g, 7.84 mmol, 4 eq.) was added to the reaction mixture, which was then stirred for 96 h at 25 °C. The reaction mixture was diluted with 50 mL of DCM and subsequently washed with 0.5% HCl (40 mL × 3), saturated NaHCO_3_ solution (40 mL × 3), and saturated NaCl solution (40 mL × 3). The organic layer was dried over anhydrous MgSO_4_ and concentrated under vacuum and then precipitated in diethyl ether to give the product as a yellow powder (7.87 g, yield 90.3%). The ^1^H NMR spectrum (400 MHz, CDCl_3_) is shown in Figure S5.

**Synthesis of P[CL*_n_*-*b*-(OEGMA*_x_*-*co*-NHSMA*_y_*)]**

NHS active ester-containing copolymers poly[caprolactone*_n_*-*b*-(oligo ethylene glycol monomethyl ether methacrylate*_x_*-*co*-*N*-succinimidyl methacrylate*_y_*)], P[CL*_n_*-*b*-(OEGMA*_x_*-*co*-NHSMA*_y_*)] was obtained by using OEGMA (average molecular weight about 452 g/mol) and *N*-succinimidyl methacrylate (NHSMA) as monomers, α, α'-azobisisobutyronitrile (AIBN) as initiator and PCL_n_-CTA as a chain transfer agent to process the reversible addition-fragmentation chain transfer (RAFT) living radical polymerization. P[CL*_n_*-*b*-(OEGMA*_x_*-*co*-NTAMA*_y_*)] was subsequently obtained by mixing the NHS active ester-containing copolymer and NTA-NH_2_ in dimethyl sulfoxide (DMSO). The reaction feed mass ratio is [PCL-CTA]/[OEGMA]/[NTAMA] = 1:2:1 to ensure a suitable hydrophilic and hydrophobic ratio to form nanoparticles. PCL-CTA (100 mg, 2.3×10^-2^ mmol, 1.0 eq), OEGMA (200 mg, 0.44 mmol, 20 eq), and NHSMA (55.5 mg, 0.30 mmol, 13.4 eq) were charged into an oven-dried vial containing 1.5 mL *N*, *N*-dimethylformamide (DMF). A stock solution of 10 mg/mL AIBN was prepared in DMF, and 90 μL of the solution (containing AIBN 0.9 mg, 5.5×10^-3^ mmol, 0.25 eq.) was added to the reaction solution after all solids dissolved. The solution was degassed using at least three freeze-pump-thaw cycles, back-filled with N_2_, and placed in a preheated oil bath at 70 °C. After 3 h, the polymerizations were quenched by submerging them into liquid nitrogen and exposing the reaction solutions to air. Purification was achieved by precipitation in diethyl ether at least three times. The ^1^H NMR spectrum (400 MHz, DMSO-*d*_6_) is shown in Figure S6. The purified copolymers' final molar and mass composition were determined by the relative integration of resonances corresponding to each monomer.

**Scheme S1.** Synthetic route of **P[CL*_n_*-*b*-(OEGMA*_x_*-*co*-NTAMA*_y_*)]**.

**Figure S1.** ^1^H NMR spectra of compound **1** in DMSO-*d*_6_.

**Figure S2.** ^1^H NMR spectra of NTA-NH_2_ in D_2_O.

**Figure S3.** ^1^H NMR spectra of PCL-OH in CDCl_3_.

**Fig. S4** GPC profiles of PCL-OH.

**Figure S5.** ^1^H NMR spectra of PCL-CTA in CDCl_3_.

**Figure S6.** ^1^H NMR spectra of (P[CL*_n_*-*b*-(OEGMA*_x_*-*co*-NHSMA*_y_*)]) in DMSO-*d*_6_.

**Figure S7.** ^1^H NMR spectra of (P[CL*_n_*-*b*-(OEGMA*_x_*-*co*-NTAMA*_y_*)]) in DMSO-*d*_6_.


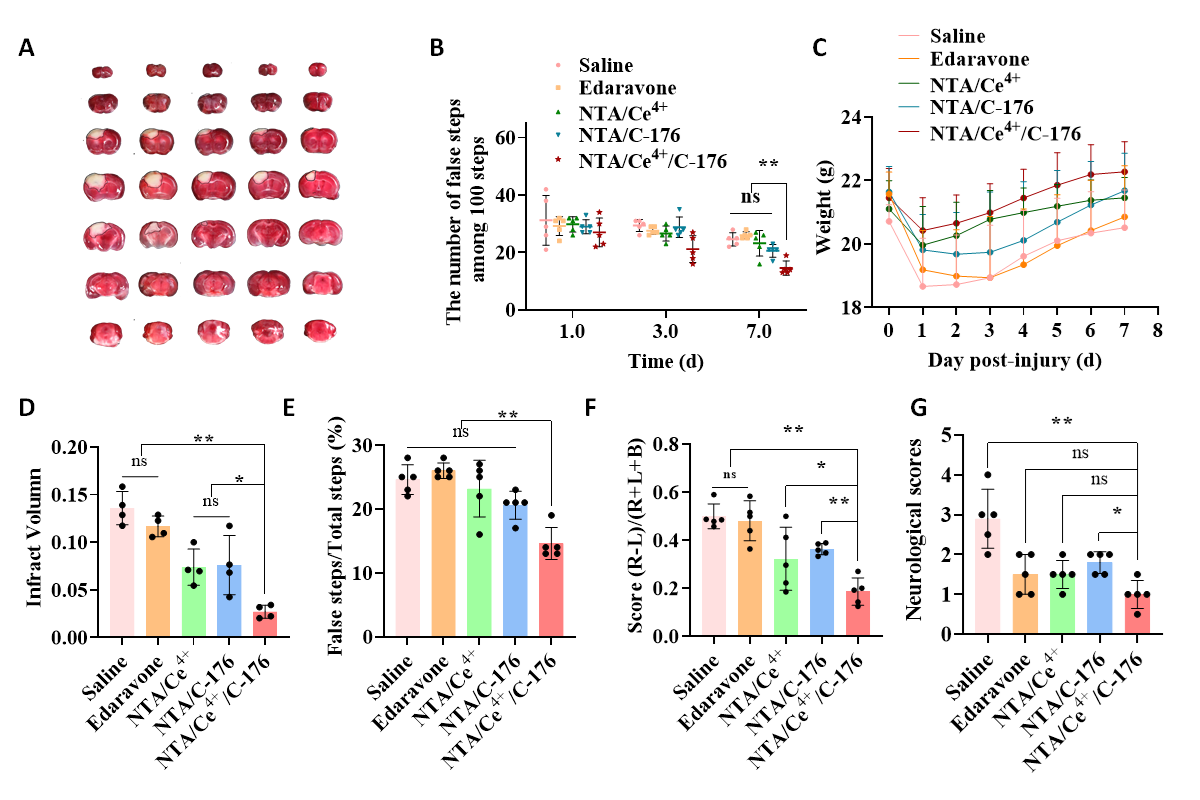


**Figure S8. Effect of NTA/Ce^4+^/C-176 on brain infarct volume and functional motor recovery after stroke.** (A) Representative images of the TTC-stained brain slice after 7 d post-stroke. The normal tissue was stained red, while the injured tissue was unstained white. (B) Body weight before cerebral infarction (d 0) and after cerebral infarction with different treatments (n = 5). (C) Grid test on 1, 3, and 7 d after stroke. (n = 5). (D) Infarct volume of the TTC-staining at 7 d post-stroke (n = 4). (E) Grid test on 7 d after stroke (n = 5). (F) Cylinder test on 7 d after therapy (n = 5). (G) Neurological scores on 3 d after therapy (n = 5). **P* < 0.05, ***P* < 0.01, ns means no significant difference.


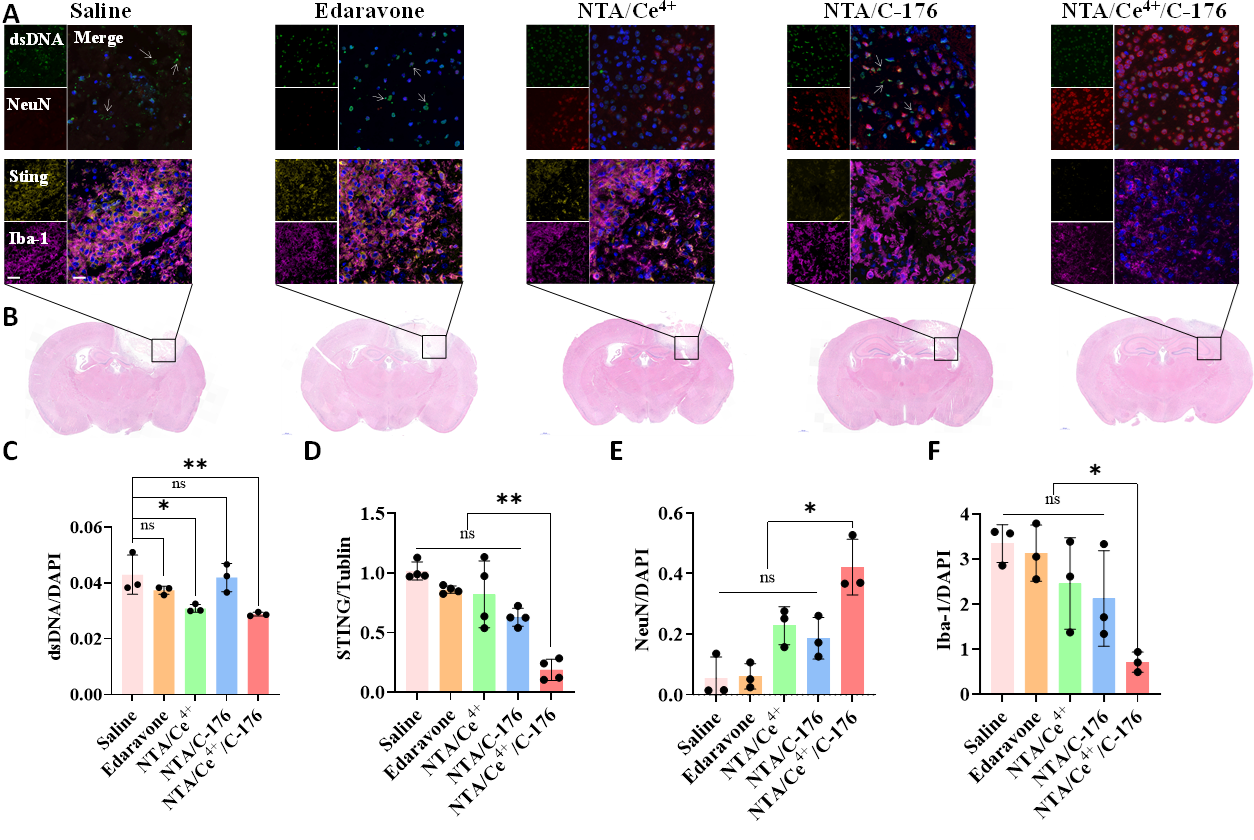


**Figure S9**. **NTA/Ce^4+^/C-176 decreased neuroinflammation and increased neurogenesis *in vivo*.** (A) Immunofluorescence of dsDNA (injury markers, green), NeuN (neuron markers, red), DAPI (nucleus markers, blue), STING (injury markers, yellow), Iba-1 (microglia markers, purple). The white arrows indicated dsDNA-positive area (injury); scale bar = 20, 40 μm). (B) The corresponding representative immunohistochemical brain slices of the immunofluorescence pictures (scale bar = 500 μm). (C) Quantitative analysis of relative dsDNA levels (n =3), STING levels in (D) (n = 4). NeuN levels in (E) and Iba-1 in (F) (n = 3) normalized to nuclei, respectively. **P* < 0.05, ***P* < 0.01, ns means no significant difference.


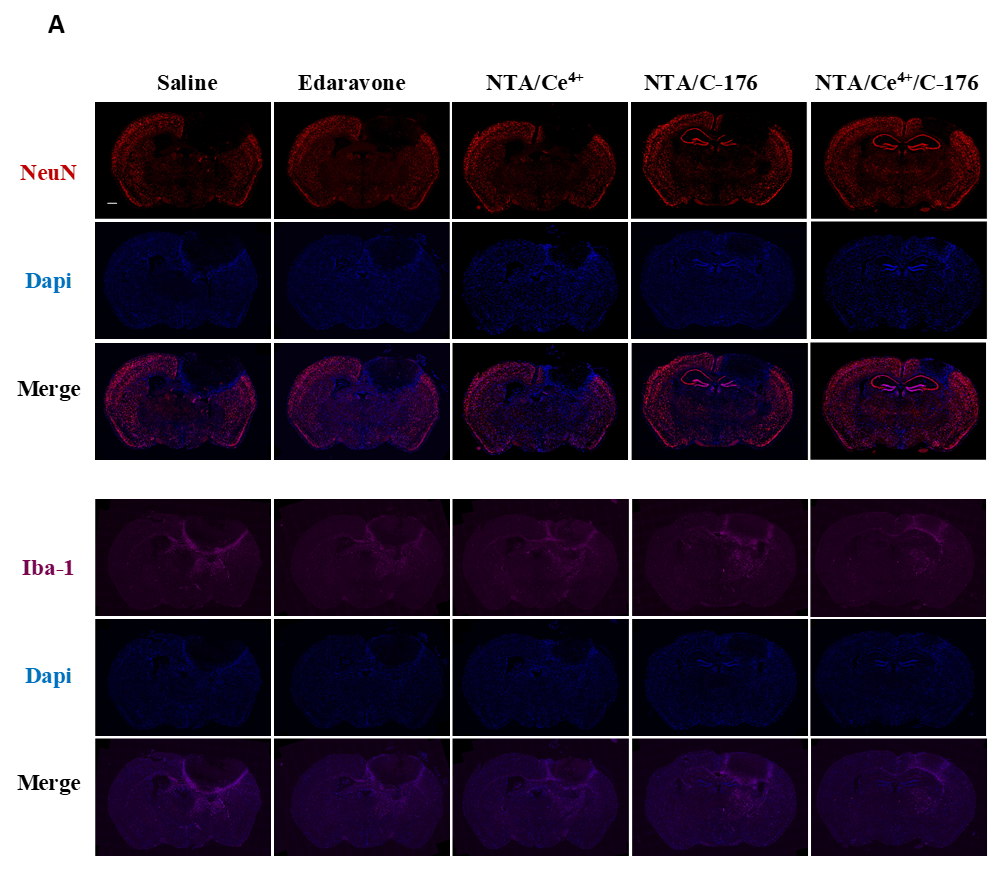
**Figure S10.** (A) Immunofluorescence of the brain at 7 d post-injection with NeuN (neurons, red), Iba-1 (microglia, purple), and DAPI (nuclei, blue); scale bar = 500 µm.

**References**

[1] Z. Chen, H. Ji, C. Liu, et al., A Multinuclear Metal Complex Based DNase-Mimetic Artificial Enzyme: Matrix Cleavage for Combating Bacterial Biofilms, Angew. Chem. Int. Ed., 55(36) (2016) 10732-10736.

[2] R.C. Pratt, B.G.G. Lohmeijer, D.A. Long, et al., Triazabicyclodecene:  A Simple Bifunctional Organocatalyst for Acyl Transfer and Ring-Opening Polymerization of Cyclic Esters, J. Am. Chem. Soc., 128(14) (2006) 4556-4557.
